# Vibration-Based Diagnostics of Rolling Element Bearings Using the Independent Component Analysis (ICA) Method

**DOI:** 10.3390/s25237371

**Published:** 2025-12-04

**Authors:** Dariusz Mika, Jerzy Józwik, Alessandro Ruggiero

**Affiliations:** 1 The Institute of Technical Sciencesand Aviation, The University College of Applied Sciences in Chelm, 22-100 Chełm, Poland; 2 Faculty of Mechanical Engineering, Lublin University of Technology, 20-618 Lublin, Poland; j.jozwik@pollub.pl; 3 Department of Industrial Engineering, University of Salerno, 84084 Fisciano, Italy

**Keywords:** blind source separation, independent component analysis, rolling bearing fault, diagnostic, sensors

## Abstract

**Highlights:**

**What is the main focus of this study?**

- The study focuses on applying Independent Component Analysis (ICA), a blind source separation technique, for vibration-based fault diagnosis of rolling element bearings.

**Which method was implemented for ICA?**

- A geodesic ICA algorithm based on Lie group optimization was implemented, ensuring stable convergence and accurate signal separation.

**How does the proposed approach improve fault detection?**

- It facilitates the extraction and enhancement of fault-related signal components, improving the detectability of characteristic defect frequencies such as BPFI and BPFO.

**What new diagnostic indicator was introduced?**

- The Locally Normalized Fault Frequency Amplitude (LNFFA) was introduced to assess the presence of specific bearing faults in localized frequency bands.

**How was the method validated?**

- Experimental results from a rotor–bearing test rig with simulated defects demonstrated clear enhancement and separation of diagnostic indicators after ICA processing compared to original signals.

**Abstract:**

This manuscript presents a study on the application of blind source separation (BSS) techniques, specifically the Independent Component Analysis (ICA) method, for the detection and identification of localized faults in rolling element bearings. Bearing defects typically manifest as distinct harmonics of characteristic fault frequencies, accompanied by modulation sidebands in the vibration signal spectrum. The accurate extraction and isolation of these components are crucial for reliable fault diagnosis, particularly in systems where multiple vibration sources overlap. In this work, a linear ICA algorithm was applied to vibration signals acquired from a simplified rotating machinery setup designed to emulate common bearing fault conditions. The study investigates the effect of ICA-based signal decomposition on the statistical distribution of selected diagnostic indicators and evaluates its ability to enhance the detectability of fault-related components. The experimental results demonstrate that the application of ICA significantly improves the separation of vibration sources, leading to a more distinct representation of fault signatures. The findings confirm the effectiveness of blind source separation methods in vibration-based diagnostics and highlight the potential of ICA as a complementary tool for improving the accuracy and robustness of bearing fault detection systems in rotating machinery.

## 1. Introduction

Rolling element bearings (REBs) are among the most widely used mechanical components in rotating machinery, being essential wherever rotary motion occurs, such as in shafts, wheels, turbines, and gear assemblies. These elements are frequently subjected to significant mechanical loads, which must be transmitted efficiently through the bearing structure. Consequently, bearings are critical components whose failure can lead not only to machine downtime and costly damage but also to potential safety hazards. The early detection of bearing faults is therefore a key aspect of predictive maintenance, aimed at preventing catastrophic failures and minimizing financial losses.

Due to their extensive use across a broad range of industrial applications, the condition monitoring and fault diagnosis of rolling bearings have been the subjects of intensive research over the past few decades. As a result, this field is now well established and supported by a variety of advanced diagnostic methodologies [1].

Classical techniques for rolling bearing fault diagnosis based on vibration signals primarily rely on analyses in the time, frequency, and time–frequency domains. Time-domain indicators such as the root mean square, kurtosis, and crest factor have traditionally been used to identify impulsive components caused by localized defects [1,2,3]. Recent studies [4,5] have enhanced these methods by introducing adaptive thresholding and data normalization, improving sensitivity under varying load and speed conditions. However, time-domain analysis alone is often insufficient, particularly when the signal-to-noise ratio is low or when multiple fault sources overlap.

Frequency-domain analysis, particularly using the Fast Fourier Transform, enables the identification of characteristic fault frequencies associated with bearing elements such as the inner race, outer race, cage, and rolling elements [6,7]. Power spectral density analysis remains a fundamental diagnostic tool, offering a clear visualization of fault harmonics and modulation sidebands. Nevertheless, purely frequency-domain methods are limited when diagnosing nonstationary signals, where fault characteristics evolve over time [8]. To address these limitations, techniques such as cepstrum analysis and spectral kurtosis have been employed to enhance fault detectability [9,10]. Another approach involves time–frequency methods such as the Short-Time Fourier Transform, Wigner–Ville distribution, and wavelet transform (WT) [11]. In particular, WT provides a favorable trade-off between time and frequency resolution and facilitates multiresolution decomposition, making it especially suitable for analyzing nonstationary vibration signals [12].

Another important development direction is cyclostationary analysis, which exploits the periodicity of bearing fault-related signals in higher-order statistics, such as the cyclic spectral correlation function [13]. Furthermore, envelope analysis, based on Hilbert transform-based demodulation, remains one of the most effective techniques for identifying characteristic fault frequencies, especially for early defect detection [14]. Classical bearing diagnostic methods are described in more detail in Section 2 of this manuscript.

To improve the extraction of weak bearing fault features masked by noise and interference, researchers have developed several advanced signal processing techniques, including Empirical Mode Decomposition [15], Ensemble Empirical Mode Decomposition, Variational Mode Decomposition [16,17], and the Synchrosqueezed Wavelet Transform [18]. These adaptive decomposition methods enable the analysis of complex vibration signals without prior knowledge of basis functions, offering higher robustness in practical applications.

With the increasing availability of large vibration datasets and advances in computational power, machine learning (ML) techniques have gained growing significance in bearing fault diagnosis. Traditional ML approaches, such as Support Vector Machines, k-Nearest Neighbors, Artificial Neural Networks, decision trees, and random forests, have been successfully applied to classify fault types based on manually engineered statistical and spectral features [19,20,21,22]. Feature selection and dimensionality reduction methods such as Principal Component Analysis and Linear Discriminant Analysis are often applied to enhance model generalization [23,24]. However, these methods largely depend on feature engineering, which may limit their adaptability and generalization performance under varying operating conditions.

To overcome the limitations of traditional ML approaches, deep learning has become a transformative tool in rolling bearing diagnostics [25,26]. Convolutional Neural Networks (CNNs) have demonstrated exceptional capabilities in capturing spatial patterns from time–frequency representations such as spectrograms and wavelet scalograms [27,28]. Recurrent Neural Networks and Long Short-Term Memory (LSTM) architectures have been used to model temporal dependencies and capture the evolution of faults over time [29,30]. More recently, one-dimensional CNNs and transformer-based models [31,32] have achieved high diagnostic accuracy under variable speed and load conditions. Transfer learning and domain adaptation techniques [33,34] are increasingly utilized to enhance model robustness when training data from the target machine are scarce.

To address the limitations of individual approaches, hybrid methods combining advanced signal processing and AI-based classifiers have emerged [35,36]. These models integrate signal processing techniques (e.g., wavelet transform, envelope analysis) with deep neural architectures, demonstrating superior robustness under noisy and nonstationary environments [37,38]. Furthermore, transfer learning and domain adaptation strategies are being increasingly explored to mitigate data imbalance and improve model generalization across different machines and operational environments [39,40].

Recent studies emphasize the integration of physics-informed AI models, federated learning, and edge computing to enable real-time fault detection in Industrial Internet of Things (IIoT) environments [41,42,43]. Despite significant progress, key challenges remain, including the interpretability of deep models, the scarcity of labeled data, and ensuring the robustness of diagnostic systems under variable operating conditions.

Typical bearing failures include corrosion of the inner and outer races, pitting due to material fatigue, and cage defects. Such faults give rise to impulsive vibration responses that excite high-frequency resonances in the bearing structure. The nature and amplitude of these resonances depend on several parameters, including the rotational speed of the shaft, bearing geometry, type of load, and fault location. Each fault type is associated with characteristic frequencies observable in the vibration spectrum. These characteristic defect frequencies are defined as follows: BPFI (Ball Pass Frequency of the Inner Race), BPFO (Ball Pass Frequency of the Outer Race), BSF (Ball Spin Frequency), and FTF (Fundamental Train Frequency). These frequencies are functions of the rotational speed and bearing geometry, and for the case of a stationary outer race, they can be determined using the standard relationships (1–4) [1]:

Ball pass frequency on outer race:

(1) $$BPFO=\frac{nf_{r}}{2}\left(1-\frac{d}{D}cos\theta \right)$$

Ball pass frequency on inner race:

(2) $$\mathrm{BPF}I=\frac{nf_{r}}{2}\left(1+\frac{d}{D}cos\theta \right)$$

Fundamental train frequency (cage speed):

(3) $$FTF=\frac{f_{r}}{2}\left(1-\frac{d}{D}cos\theta \right)$$

Ball spin frequency:

(4) $$BSF=\frac{D}{2d}\left(1-{\left(\frac{d}{D}cos\theta \right)}^{2}\right)$$

where

$f_{r}$ is the shaft rotational frequency;
n is the number of rolling elements;
d is the diameter of a rolling element;
D is the pitch diameter of the bearing;
θ is the contact angle between the rolling element and the raceway.

The contact between rolling elements and a raceway defect induces high-frequency resonances within the bearing structure. These resonances appear in the upper part of the vibration signal spectrum. The characteristic defect frequencies defined earlier are only visible in the envelope spectrum of this signal. The envelope can be obtained by performing a Hilbert transform of the vibration signal or via complex demodulation (forming the analytical signal).

Most advanced REB diagnostic techniques are based on identifying these characteristic frequencies within the vibration spectrum. For this purpose, specially constructed diagnostic indicators are used, whose values determine the presence or absence of a particular fault. Examples of such indicators and their influence on fault identification efficiency are presented in [44]. The general idea behind using these indicators is that the “stronger” the indicator is in the presence of a fault, the more effective the fault identification becomes. The term “stronger indicator” refers to a diagnostic parameter whose values, when a fault is present, identify the fault with higher probability.

The main idea of this study is to enhance the performance of such diagnostic indicators by applying blind source separation (BSS) methods. These techniques can be generally defined as data-driven, unsupervised methods for detecting hidden features within signals. Developed for more than four decades, they are now well understood theoretically and widely applied across many fields of science and engineering—for example, in biomedical signal analysis (ECG, EEG, MEG, fMRI), audio–video technology, telecommunications [45], economics, and even astrophysics. They can be applied wherever the analyzed system—mechanical, electrical, economic, or biological—produces signals that are mixtures of several underlying components, while the useful information for analysis lies in the source signals themselves. The objective of BSS methods is therefore to separate or extract the source signals.

The application of the ICA method in rolling bearing diagnostics can represent a significant methodological innovation, enabling the overcoming of one of the key limitations of classical vibration signal analysis techniques—namely, the difficulty in isolating fault-related components from complex, overlapping signal sources and noise. Unlike traditional methods, which require prior knowledge of fault characteristics or assume signal linearity and stationarity, ICA belongs to the class of blind source separation methods and enables unsupervised extraction of statistically independent source components from multichannel vibration measurements. Consequently, this technique can be particularly useful for diagnosing complex and coexisting faults (known as compound faults).

From an analytical perspective, ICA improves both interpretability and the signal-to-noise ratio of diagnostic indicators derived from vibration signals, as demonstrated in Section 5 of this manuscript. By decomposing the signal into independent components, the method effectively separates those parts corresponding to vibrations induced by bearing defects (e.g., BPFI, BPFO), while suppressing the influence of other system elements (e.g., gears, shafts, structural resonances). As a result, applying ICA as a preprocessing step before conventional envelope analysis leads to an “enhancement” of modulation signals associated with faults, significantly improving the sensitivity and accuracy of characteristic frequency and sideband identification. Such a hybrid combination of ICA and envelope analysis therefore enables more effective fault detection under low signal-to-noise ratio (SNR) conditions.

The motivation for this study was to examine whether these methods could effectively separate or extract fault-related syndromes (sources) from the complex mixed vibration signal measured from a mechanical system.

This manuscript is organized as follows. The section “Vibration-Based Fault Identification Methods” presents the fundamental techniques used to identify fault-related features based on measured vibration signals. The section “Principles of the ICA Method” introduces the basic linear model of the Independent Component Analysis (ICA) method. The subsection entitled “Geodesic ICA Algorithm Based on Toral Decomposition” describes the geodesic ICA algorithm employed in the experimental part of this work. Examples of fault identification indicators for REBs are presented in the subsection “Fault Identification Indicators”. The proposed research methodology is discussed in the section “Proposed Methodology”. Finally, the section “Results and Discussion” presents the results of the experiment involving the application of the ICA method to mixed vibration signals obtained from a rotor machine with simulated bearing defects. The influence of applying BSS techniques, particularly ICA, on the distribution of the adopted fault identification indicator is also analyzed.

## 2. Classical Vibration-Based Fault Identification Methods

Classical vibration-based methods for identifying rolling bearing faults can be classified into five main groups [46]:

- Time-domain analysis methods;
- Frequency-domain analysis methods;
- Time–frequency-domain analysis methods;
- Envelope analysis of vibration signals;
- Cyclostationary analysis.

**Time-Domain Analysis Methods**: In approaches based on time-domain signal analysis, bearing fault diagnosis involves identifying characteristic statistical features that distinguish healthy bearings from damaged ones. These parameters can be determined in various ways, such as by counting impact impulses [47], calculating the root mean square (RMS) value per shaft rotation, and determining the maximum signal amplitude. Other indicators, such as the crest factor or kurtosis, are also commonly used, as they provide information about irregular changes in the vibration signal [48,49,50]. Statistical parameters in the time domain—including mean value, standard deviation, peak value, crest factor, and kurtosis—serve as trend indicators and are widely applied for the early detection of incipient bearing defects.

**Frequency-Domain Analysis Methods**: In vibration analysis techniques, the Fourier transform is often used to convert a time-domain signal into its frequency-domain representation. This typically yields a power spectrum, with computations efficiently performed using the Fast Fourier Transform algorithm, significantly reducing processing time. This topic has been extensively discussed in the literature, with a comprehensive overview provided in the monograph “Frequency Analysis” [51].

In the context of rolling bearing diagnostics, spectral analysis is not usually employed as an independent fault detection method. Rather, it serves as a comparative tool that facilitates tracking changes within specific frequency bands [52,53]. Based on these observations, frequency ranges particularly sensitive to disturbances can be identified and subjected to more advanced diagnostic techniques, such as envelope detection.

Time–Frequency-Domain Analysis Methods: These methods are based on the simultaneous analysis of vibration signals in both the time and frequency domains. The simplest approach is the Short-Time Fourier Transform (STFT), which provides a joint time–frequency representation. More advanced techniques belong to the Cohen’s class [11], including the Wigner–Ville Distribution (WVD) and its various modifications. Another widely used approach is based on the wavelet transform [54,55], which enables flexible analysis of nonstationary signals.

However, both STFT and WVD have limitations that affect their practical application in diagnostics. For STFT, the issue lies in its fixed time and frequency window widths—improving resolution in one domain necessarily reduces it in the other, since the product of time and frequency resolutions remains constant [54]. On the other hand, WVD may produce negative energy levels and cross-terms that complicate interpretation [56]. These limitations have driven the development of alternative approaches, among which wavelet-based methods are particularly effective, offering a better trade-off between time and frequency resolution.

**Envelope Analysis of Vibration Signals**: The envelope analysis method for rolling bearing vibration signals [57] is based on the assumption that, when a localized surface defect on a bearing element comes into contact with another component under load, a short-duration impulse is generated. The duration of this event is extremely brief compared to the interval between successive impulses, causing the signal energy to spread across a wide frequency range. Consequently, such an impact excites numerous resonances both within the bearing itself and in its surrounding structure.

To enable further analysis, the envelope is extracted from the original time-domain signal using the Hilbert transform, yielding the so-called analytic signal. In practice, envelope analysis is particularly useful because raw vibration signals are often difficult to interpret directly. The procedure involves demodulating the analytic signal through band-pass filtering, which enhances the clarity of resonant components and helps to isolate bearing-related features from other noise sources.

In many cases, the squared envelope is also analyzed to further improve the signal-to-noise ratio. The principal advantage of envelope analysis lies in its ability to distinctly extract fault-specific characteristic frequencies and their accompanying modulation sidebands, making it a far more effective diagnostic technique than conventional approaches.

**Cyclostationary Analysis**: Vibration signals can generally be classified into two main categories: stationary and nonstationary. A signal is considered stationary when its statistical properties remain constant over time, whereas nonstationary signals exhibit time-varying characteristics. For analyzing such signals, methods based on the autocorrelation function are often employed. A special class of nonstationary signals are cyclostationary signals, whose autocorrelation function varies periodically. Applying a two-dimensional Fourier transform to the autocorrelation function yields the spectral correlation function (SCF).

SCF analysis serves as a tool for distinguishing between stationary, cyclostationary, and periodic signals, thereby enabling the identification of various fault sources. Examples of this approach are presented in [58], where the authors utilized the properties of cyclostationary processes to separate modulations arising from bearing faults from those associated with gear defects.

## 3. Theoretical Foundations of the ICA-Based Fault Identification Method

### 3.1. Principles of the ICA Method

The basic linear instantaneous model of the Independent Component Analysis (ICA) method can be expressed by Equation (5) [45]:

(5) x=As

where

$x={\left(x_{1},\ldots ,x_{n}\right)}^{T}$ is the vector of mixed (observed) signals;
$s={\left(s_{1},\ldots ,s_{n}\right)}^{T}$ is the vector of source signals;
A is a constant, non-singular (invertible) n×n mixing matrix.

**Figure 1:**
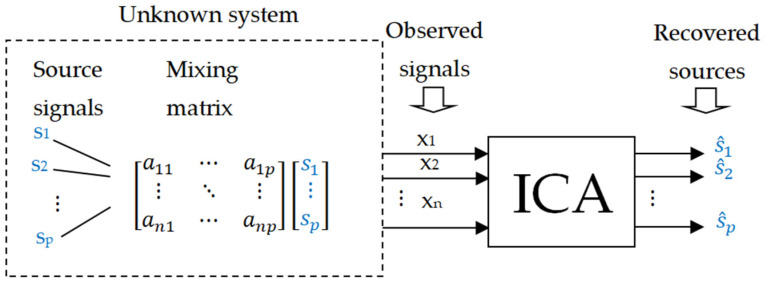
Schematic block diagram of the Independent Component Analysis.

In the ICA method (Figure 1), the unknown quantities are the mixing matrix A and the vector of source signals s, whereas the only available data are the mixed signals x, which are linear combinations of the source signals $s_{i}$.

ICA is a statistical method that estimates a set of statistically independent variables—i.e., the source signals s—and the corresponding mixing matrix (the parameters of the mixing system) solely on the basis of their mixtures x. More precisely, the goal of ICA is to find (estimate) a linear transformation $W≅A^{-1},W\in Gl\left(n\right)$, referred to as the separating or demixing matrix, given N observations of the mixed signal x. The estimators of the source signals $\hat{s}$ (hidden features or latent variables) are obtained from Equation (6):

(6) $$\hat{s}=Wx=WAs≅s$$

It can be shown that such a system can be determined only up to the scale and permutation of the source signals, which represents an inherent indeterminacy of the ICA method.

There are generally two main approaches to solving the ICA problem:

1. Algebraic methods;
2. Optimization-based methods that rely on specially designed contrast functions.

Algebraic methods involve the diagonalization or joint diagonalization of certain matrices containing cumulants of various orders. For example, in the classical JADE (Joint Approximate Diagonalization of Eigenmatrices) algorithm [59], fourth-order cumulant matrices are jointly diagonalized via cyclic application of the well-known Givens (Jacobi) rotations from linear algebra.

The second group of ICA algorithms aims to find a global extremum of a contrast function, which serves as a measure of the statistical independence of the separated signals. These are optimization methods, which can generally be classified as gradient-based, Newton, quasi-Newton, and conjugate-gradient methods.

Gradient-based methods are relatively simple and computationally inexpensive optimization techniques in which the search direction is defined by the gradient of the contrast function. However, they exhibit only linear convergence. A classical example of a gradient-based ICA algorithm is INFOMAX [60], where the log-likelihood function is used as the contrast function.

In contrast, Newton and quasi-Newton methods incorporate second-order information about the contrast function, i.e., the Hessian matrix or its approximations, in the optimization process. Although these methods are computationally more demanding, their convergence rate is at least quadratic. A well-known example of a quasi-Newton ICA algorithm is FastICA [45].

There also exists a class of optimization algorithms with a strong theoretical foundation that exploit the geometric structure of the optimization space. In ICA, the space of separating matrices can be viewed as a differentiable manifold that also possesses group properties. Such spaces are known as Lie groups, and optimization algorithms that operate on them are referred to as Lie group algorithms or geodesic flow algorithms.

In the general ICA model (Equation (8)), the optimization space corresponds to the general linear group $Gl\left(n\right)$ of separating matrices. However, in most ICA algorithms, a preprocessing step known as whitening is applied, which decorrelates and normalizes the observed signals. In such cases, the optimization is performed on the special orthogonal group $SO\left(n\right)$, i.e., the group of orthogonal separating matrices ($W^{T}W=I$) with a unit determinant (detW=1).

Examples of efficient ICA algorithms operating on the special orthogonal group $SO\left(n\right)$, or on the unitary group $U\left(n\right)$ in the complex case, can be found in [61,62,63,64,65]. The ICA algorithm applied in the experimental study presented in Section 5 also belongs to the class of geodesic flow algorithms and will be discussed in more detail in the following section.

The ICA model can be extended to nonlinear forms when the assumption of linearity is not valid, for example, when observed signals are distorted by nonlinear processes in the measurement path. However, in most practical engineering applications, including vibration analysis, the linear ICA model is sufficient to achieve effective signal separation.

In the context of vibration-based diagnostics, ICA facilitates the decomposition of complex, mixed vibration signals into statistically independent components. These components may correspond to different physical sources of vibration, such as unbalance, misalignment, or bearing defects. By isolating components associated with bearing faults, ICA enhances the clarity of defect-related features in the signal and improves the performance of diagnostic indicators.

### 3.2. Geodesic ICA Algorithm Based on Toral Decomposition

In the experimental study presented in Section 5, a linear instantaneous ICA algorithm of the geodesic flow type was employed. The optimization space, i.e., the space of separating matrices, is modeled as the Lie group $SO\left(n\right)$. The corresponding Lie algebra $so\left(n\right)$ of this group—representing the tangent vector space at the identity element—is the set of antisymmetric matrices [66].

In this case, the optimization scheme is multiplicative, meaning that the next iteration of the separating matrix $W_{k+1}$ is obtained by multiplying the previous one $W_{k}$ by an orthogonal rotation matrix $R_{k}\in SO\left(n\right)$ which is generated through the exponential mapping of a properly constructed element of the Lie algebra $so\left(n\right)$, i.e., the antisymmetric gradient of the contrast function in the following form (7):

(7) $$\Omega =W_{k}^{T}\nabla_{W}f-\nabla_{W}^{T}fW_{k}\in so\left(n\right)$$

where

$\nabla_{W}f$ is the Euclidean gradient of the contrast function evaluated at $W_{k}$.

During the exponentiation step, a Schur decomposition of the antisymmetric gradient matrix is applied, introducing torus geometry into the optimization space.

Real Schur decomposition of a normal matrix $A=R^{n\times n}$ is presented in the block-diagonal form (8):

(8) $$Q^{T}AQ=\left[\begin{array}{cccc} R_{11} & 0 & \ldots & 0\\ 0 & R_{22} & \ldots & 0\\ \vdots & \vdots & \ddots & \vdots \\ 0 & 0 & \ldots & R_{mm} \end{array}\right]$$

where $Q=R^{n\times n}$ is the orthogonal matrix and each $R_{ii}$ is either a 1-by-1 or a 2-by-2 matrix. Each 1-by-1 block corresponds to a real eigenvalue of A and each 2-by-2 block to a complex eigenvalue of A and has the form $\left(\begin{array}{cc} a & b\\ -b & a \end{array}\right)=\alpha \left(\begin{array}{cc} c & s\\ -s & c \end{array}\right)$, where α∈R and matrix $\left(\begin{array}{cc} c & s\\ -s & c \end{array}\right)$ is an orthogonal (rotating) matrix.

In the case of antisymmetric gradient matrices Ω, whose eigenvalues are purely imaginary, the Schur form supersedes the form (9):

(9) $$\Omega =Q\left(\begin{array}{ccc} \Phi_{1} & \ldots & 0\\ \vdots & \ddots & \vdots \\ 0 & \ldots & \Phi_{m} \end{array}\right)Q^{T}$$

where $Q\in SO\left(n\right)$ and $\Phi_{i}=\left(\begin{array}{cc} 0 & \varphi_{i}\\ -\varphi_{i} & 0 \end{array}\right)$ are antisymmetric 2×2 matrices with scalar parameter $\varphi_{i}\in R$.

Since the relation $\exp\left(Q^{T}\Omega Q\right)=Q^{T}\exp\left(\Omega \right)Q$ is satisfied, the exponentiation of the matrix Ω results in the matrix R of the form:

(10) $$R=\exp\Omega =Qdiag(R_{1},\ldots ,R_{m})Q^{T}$$

where $R_{i}=\left(\begin{array}{cc} cos\varphi_{i} & sin\varphi_{i}\\ -sin\varphi_{i} & c{os\varphi }_{i} \end{array}\right)$ are the rotating matrices. The overall computational cost is thus reduced to evaluating simple trigonometric functions—sine and cosine—of scalar parameters obtained from the Schur decomposition, and the Schur decomposition itself can be performed highly efficiently using numerical methods [67].

The iterative optimization scheme of the algorithm can be expressed as in Equation (11):

(11) $$W_{k+1}=W_{k}R_{\Omega_{k}}=W_{k}exp(-\mu \Omega_{k})$$

where

$\Omega_{k}$ is an antisymmetric gradient in the *k*-th iteration;
μ is the iteration step size.

As a result, the algorithm exhibits high computational efficiency, fast convergence, and numerical stability, while maintaining excellent separation performance [61,63].

The following Algorithm 1 summarizes the presented geodesic ICA algorithm in pseudo-code form.
**Algorithm 1:** ICA algorithm in pseudo-code form1:**Input:** ${x\;\in R}^{n\times N}$—multichannel measured (mixed) signal2:Initialize random ${W_{0}\;\in R}^{n\times n}$Loop:3:for k = 0: minimization4:Compute the Euclidean gradient of the contrast function $\nabla_{W_{k}}f$5:Compute antisymmetric gradient $\Omega_{k}=W_{k}^{T}\nabla_{W}f-\nabla_{W}^{T}fW_{k}\in so\left(n\right)$ as in (7)6:Perform the Schur decomposition of the matrix $\Omega_{k}$ as in (9)7:Move into the direction of the negative antisymmetric gradient ${-\Omega }_{k}$ with adaptively chosen (or experimentally) step size μ: $R_{\Omega_{k}}=Qdiag\left(R_{1},\ldots ,R_{m}\right)Q^{T}$, where $R_{i}=\left(\begin{array}{cc} cos\left(-\mu \varphi_{i}\right) & sin\left(-\mu \varphi_{i}\right)\\ -sin\left(-\mu \varphi_{i}\right) & cos\left(-\mu \varphi_{i}\right) \end{array}\right)$ as in (10)8:Update: $W_{k+1}=W_{k}R_{\Omega_{k}}$, k:=k+1 as in (11). Iterate the loop 3–8 until convergence9:end10:**Output:** W and s=Wx—recovered source signals

### 3.3. Fault Identification Indicators

Vibration-based diagnostics of rolling element bearings is fundamentally associated with the effective identification of characteristic fault frequencies. The identification of these frequencies in the vibration signal spectrum requires the construction of appropriate indicators that determine the presence or absence of specific defects. The threshold values of these indicators—defining whether a fault is present—are established based on the distribution of indicator values obtained from a training dataset consisting of vibration signals measured from faulty bearings.

For single, isolated defects, it is possible to construct an indicator whose specific values directly identify the defect type, such as an inner race or outer race fault. An example of such an indicator can be found in [68]. The authors of that study introduced a relative fault frequency amplitude indicator known as the Log Ratio of Fault Amplitude (LRFA), defined as follows (12):

(12) $$LRFA=\log\left(\frac{BPFIAmplidude}{BPFOAmplidude}\right)$$

where BPFIAmplidude and BPFOAmplidude denote the amplitudes of the characteristic fault frequencies for the inner and outer race, respectively.

Based on training signals, it is possible to determine specific ranges of LRFA values corresponding to an inner race fault, outer race fault, or normal bearing operation (no defect).

However, relative indicators such as LRFA fail in cases where multiple defects occur simultaneously—for example, inner and outer race faults—since both characteristic frequencies appear in the spectrum. Under such conditions, the LRFA value may fall within the range corresponding to normal operation, leading to diagnostic ambiguity.

To overcome this limitation, fault-specific indicators can be designed. In [44], a Normalized Amplitude of Fault Frequency (NAFF) indicator was introduced, defined as follows (13):

(13) $$NAFF=\log\left(\frac{FFA}{\sum PA}\right)$$

where

FFA denotes the amplitude of the characteristic fault frequency;
∑PA represents the sum of amplitudes across the entire spectrum.

This indicator measures the relative amplitude of a selected fault frequency. However, in many cases, a substantial portion of the vibration signal’s energy is concentrated in the upper part of the spectrum. Consequently, the NAFF indicator may be less sensitive to variations in the amplitudes of characteristic fault frequencies, as it considers the total spectral energy.

In this study, a new indicator was proposed—one that evaluates the presence of characteristic fault frequencies in a more localized manner—referred to here as the Locally Normalized Fault Frequency Amplitude (LNFFA). This indicator considers only the frequency band within the range (BPFO,2·BPFO) and measures the relative amplitudes of the characteristic frequencies BPFO and BPFI with respect to that range.

The indicator takes distinct forms for outer race and inner race defects. Notably, in the case of an inner race fault, the envelope spectrum not only includes the BPFI frequency but also exhibits strong sidebands associated with the shaft rotational frequency $f_{r}$. For BPFO identification, the indicator also includes the amplitude at 2·BPFO, while for BPFI identification, it incorporates the sideband amplitudes at $\left(BPFI-f_{r}\right)$ and $\left(BPFI+f_{r}\right)$.

The indicator is defined as follows (14 and 15):

(14) $${LNFFA}_{Outer}=\log\left(\frac{{FFA}_{BPFO}+{FFA}_{2\cdot BPFO}}{\sum {PA}_{\left(BPFO,\;2\cdot BPFO\right)}}\right)$$

(15) $${LNFFA}_{Inner}=\log\left(\frac{{FFA}_{BPFI}+{FFA}_{\left(BPFI-f_{r}\right)}+{FFA}_{\left(BPFI+f_{r}\right)}}{\sum {PA}_{\left(BPFO,\;2\cdot BPFO\right)}}\right)$$

where the subscript of FFA denotes the frequency to which the amplitude refers.

This indicator is correctly defined when the condition $\left(BPFI-f_{r},BPFI+f_{r})\in (BPFO,2\cdot BPFO\right)$ is satisfied. It can be shown that, for n>2 (where n is the number of rolling elements), this condition holds if D<nd, which is valid for most standard rolling element bearings.

The LNFFA indicator described above was employed in the experimental study presented in Section 5.

## 4. Proposed Methodology

Every operating mechanical system generates a complex vibro-acoustic signal that is a superposition of signals originating from various sources. These signals are produced by individual components of the mechanical system as well as via different processes occurring within it. The occurrence of a fault in the system gives rise to a specific mechanical phenomenon that contributes an additional component to the overall vibro-acoustic signal.

From a diagnostic perspective, the extraction—or at least the enhancement—of this fault-related component can significantly improve the effectiveness of fault identification.

The methodology proposed in this manuscript involves applying the ICA method to the measured complex vibration signal to extract or amplify the fault-related component. The source signal obtained as a result of the ICA decomposition is expected to exhibit greater fault identifiability compared to the original (measured) signal.

**Figure 2:**
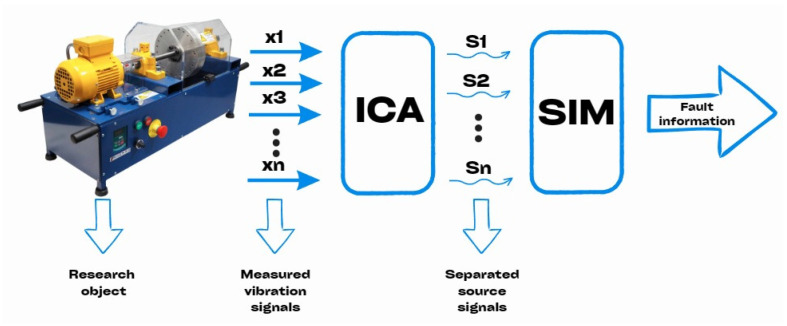
Block diagram illustrating the operation of a diagnostic expert system based on BSS and SIM.

The proposed concept is schematically illustrated in Figure 2. The measured complex vibration signals $\left\{x_{i}\right\}$, treated as a multi-channel mixed signal x, are processed using the ICA algorithm, yielding a set of source signals $\left\{s_{i}\right\}$. These extracted signals are then analyzed using Standard Identification Methods (SIMs) applied in vibration-based fault diagnosis.

At this stage, any of the diagnostic methods presented in Section 3 can be used. In the experimental study presented in the following section, the envelope analysis method was employed due to its proven effectiveness in identifying the characteristic fault frequencies of rolling bearings defined by relationships (1–4).

The proposed methodology is summarized below, outlining each signal processing step that is discussed in detail in the preceding sections and further elaborated in the following Section 5.

1. Data acquisition
  - Acquire synchronous multi-channel vibration signals (we used 2 channels) at sampling frequency $f_{s}$ (experiment: $f_{s}=6400\;Hz$) and record duration (experiment: 10 s).
2. Preprocessing
  - Remove DC offset.
  - Normalize each channel to unit variance.
  - Whitening (PCA sphering): compute covariance of the multichannel data, perform eigen decomposition, and transform data to zero-mean, unit-variance, uncorrelated signals.
3. ICA decomposition (geodesic ICA)
  - Use whitened data as input.
  - Algorithm specifics used in experiments: geodesic flow ICA with Schur decomposition at exponentiation stage; negentropy contrast approximated by ($g(x)=x^{4}$).
  - Output: separating matrix (W) and estimated source signals (s=Wx).
4. Envelope extraction (SIM stage)
  - Compute analytic signal via the Hilbert transform and obtain the envelope; compute envelope power spectrum (FFT of envelope). Use the MATLAB 2024b ‘envspectrum’ routine or equivalent.
5. Fault indicator computation (LNFFA)
  - For the envelope power spectrum compute amplitudes at target fault frequencies and sidebands.
  - Compute LNFFA as in (14–15).

## 5. Results and Discussion

**Figure 3:**
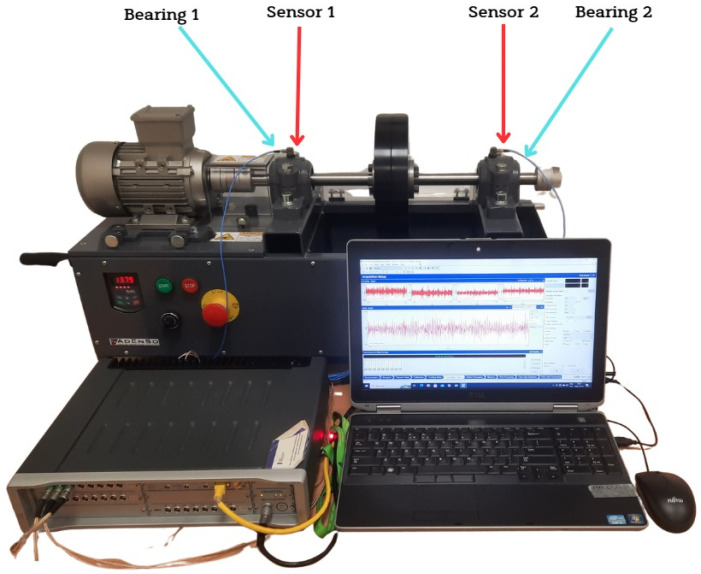
Experimental test stand.

The conducted experiment involved measuring vibration signals from a rotor machine with intentionally introduced bearing race defects, followed by the application of the geodesic ICA algorithm described in Section 4. The experimental test stand is shown in Figure 3.

The mechanical system, in the form of a rotor machine, consisted of a DC motor, a shaft supported in two ball bearings (MB ER10K (Rexnord Corporation, Valparaiso, Indiana, USA)), and a steel cylindrical load. Ball bearings were mounted in two supports—the first located near the motor and the second at the end of the shaft. Two vibration sensors were attached to the bearing housings, as shown in Figure 3. The vibration signals were recorded using a Siemens LMS Scadas Mobile signal analyzer (Siemens AG, Munich, Germany). Piezoelectric ICP accelerometers PCB 356B21 (PCB Piezotronics, Inc, Depew, NY, USA) with a sensitivity of 10 mV/g were used for vibration measurements.

Three fault conditions were introduced into the rotor machine:

1. Inner race fault of the left bearing (near the motor);
2. Outer race fault of the right bearing;
3. Simultaneous outer race fault of the left bearing and inner race fault of the right bearing.

**Figure 4:**
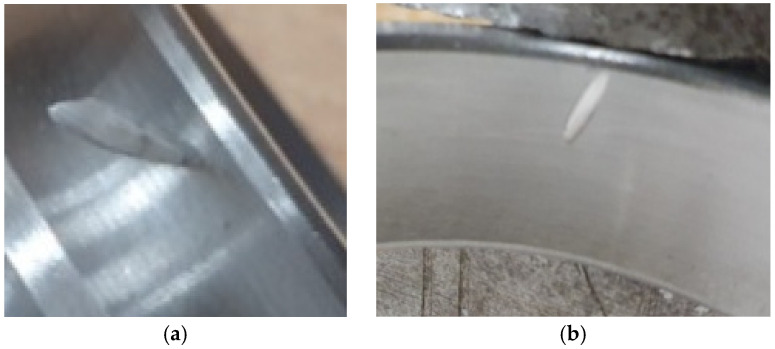
Simulated defects: (**a**) inner race fault, (**b**) outer race fault.

The race defects were simulated by introducing notches, as shown in Figure 4. Measurements were conducted at a rotational speed of approximately 3000 rpm ($f_{r}=50\;Hz$). Rotational speed was determined by adjusting the motor controller settings. The rotational speed was precisely determined in postprocessing through spectrum analysis. The sampling frequency was $f_{s}=6400\;Hz$. In total, 50 vibration signals were recorded for each of the three fault conditions, resulting in a dataset of 150 signals. Each signal had a duration of 10 s (64,000 samples).

In the blind source separation stage, several contrast functions were tested, including those based on log-likelihood and negentropy approximation. The best results were obtained using a negentropy-based contrast function with a nonlinearity of the form ${\left(x_{i}\right)}^{4}$ [45]. All further analyses were performed under these conditions.

The vibration signals measured from the two sensors were treated as a two-channel mixed signal, which served as the input to the ICA algorithm. The training dataset consisted of 100 original measured signals and 100 separated source signals obtained after applying ICA (25 per single fault type and 50 for the simultaneous fault condition). Below is an example of an estimated orthogonal separating matrix $W≅A^{-1}$:

$$W=\left(\begin{array}{cc} -0.2069 & 0.9784\\ 0.9784 & 0.2069 \end{array}\right)$$

Based on the manufacturer’s bearing geometry data and precise measurement of the shaft rotational speed, the characteristic fault frequencies BPFI and BPFO were determined. Depending on the rotational frequency $f_{r}$, these values varied within the following ranges: BPFI=147÷150 Hz, BPFO=236÷240.5 Hz. All signals were normalized prior to the SIM stage—the DC offset was removed, and the variance was scaled to 1. Envelope analysis was applied in the SIM stage. The Matlab function envspectrum was used to obtain the envelope power spectra of the signal. The Hilbert transform method was selected with standard filtering settings (a FIR filter of order 50 with passband of $\left[f_{s}/4,3f_{s}/8\right]$, where $f_{s}$ is the sampling frequency).

The fault identification indicator LNFFA described in Section 3.2 was used. The collected training signal base (200 signals) was utilized to analyze the distribution of the proposed indicator values for each fault condition and to compare these distributions for the original and ICA-separated signals.

The results for the three simulated fault conditions are presented below. For single faults (conditions 1 and 2), from each pair of recorded signals (original and ICA-separated), only the signal with the higher indicator value was used for analysis. For the simultaneous fault condition (condition 3), both signals were analyzed.

### 5.1. Inner Race Fault of the Left Bearing

**Figure 5:**
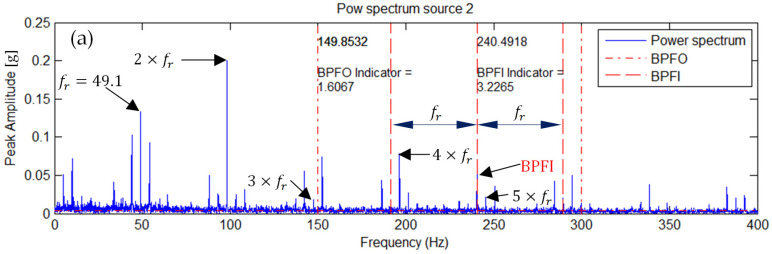

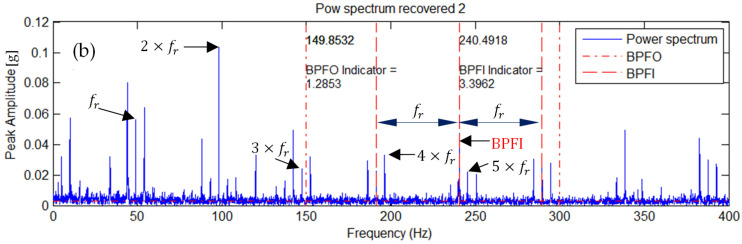
Envelope spectrum of (**a**) the measured signal and (**b**) the ICA-separated source signal for a single inner race fault.

Figure 5 shows the envelope spectrum of a representative vibration signal—both the original and the ICA-separated one. Vertical lines indicate the characteristic fault frequencies BPFI corresponding to the inner race defect, along with the sidebands $\left(BPFI-f_{r}\right)$ and $\left(BPFI+f_{r}\right)$ related to the rotational frequency $f_{r}$, as well as the outer race fault frequency BPFO.

Alongside these frequency lines, the corresponding fault identification indicator values for BPFI and BPFO are shown.

In the envelope spectrum of the ICA-separated source signal, a significant enhancement of the BPFI frequency and its sidebands $\left(BPFI-f_{r}\right)$ and $\left(BPFI+f_{r}\right)$ is clearly visible. This results in an increase in the BPFI indicator value, while the BPFO indicator value decreases. This selective amplification results in an increase in the BPFI fault indicator value from 3.2265 to 3.3962, demonstrating improved detectability of the inner race defect.

### 5.2. Outer Race Fault of the Right Bearing

**Figure 6:**
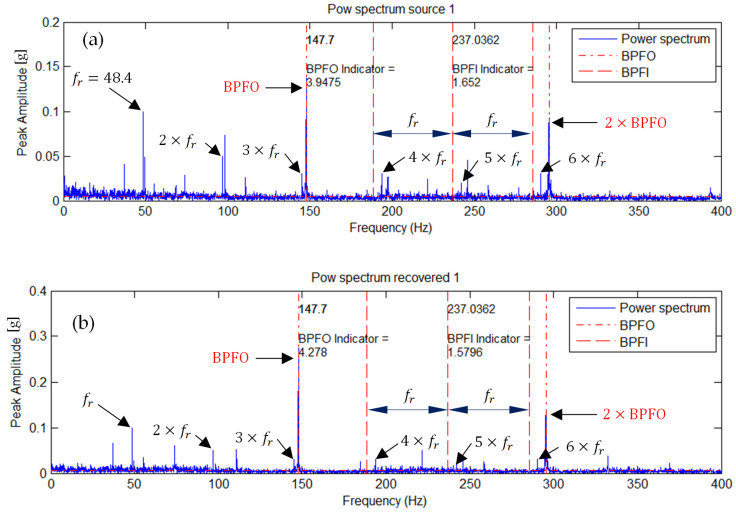
Envelope spectrum of (**a**) the measured signal and (**b**) the ICA-separated source signal for a single outer race fault.

A similar effect is observed for the outer race defect. Figure 6 illustrates the envelope spectra corresponding to this condition, where the BPFO fault component becomes dominant in the ICA-separated signal. The BPFO indicator value increased from 3.9475 (original) to 4.2780 (after ICA), confirming that ICA enhances the discriminative power of fault-related features by isolating the relevant source component.

### 5.3. Simultaneous Outer Race Fault of the Left Bearing and Inner Race Fault of the Right Bearing

**Figure 7:**
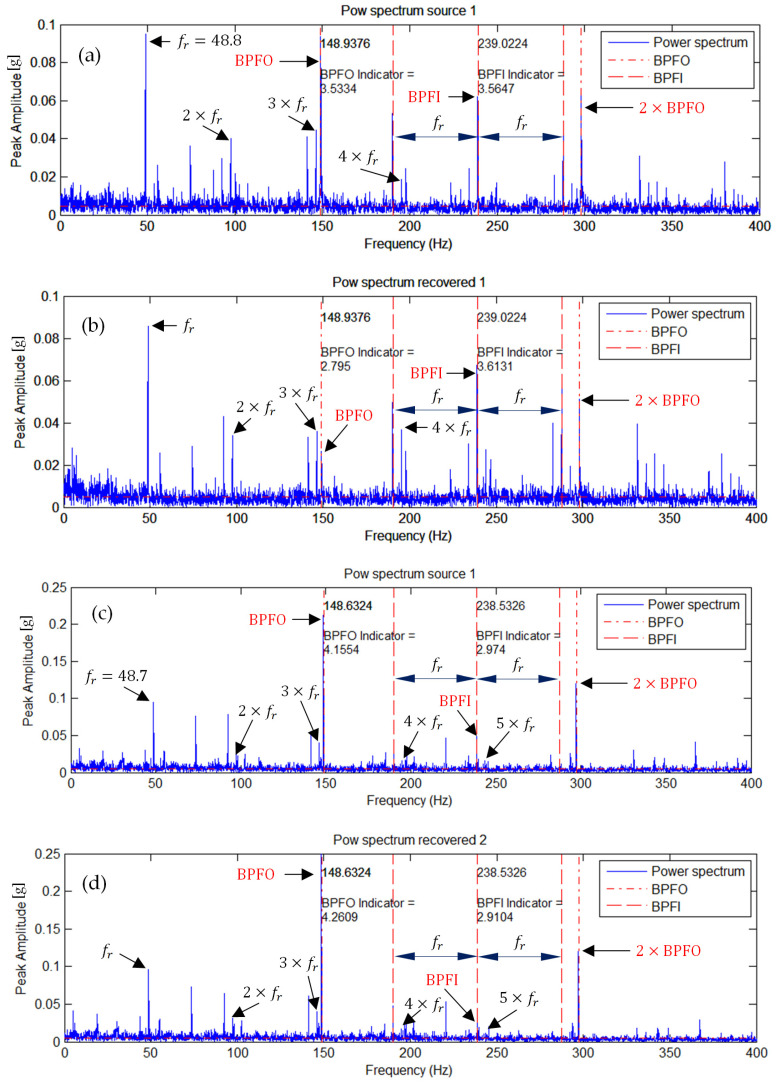
Envelope spectra of representative measured and ICA-separated source signals for the simultaneous fault condition: (**a**) measured signal 1, (**b**) source signal 1 after ICA, (**c**) measured signal 2, and (**d**) source signal 2 after ICA.

Figure 7 shows the envelope spectra of representative measured (original) and ICA-separated source signals. In the spectrum corresponding to the ICA-separated signal (Figure 7b), a strengthening of the BPFI spectral components and a noticeable attenuation of the BPFO components are observed. In contrast, the spectrum of the original signal (Figure 7a) exhibits both BPFI and BPFO frequencies simultaneously.

Similarly, in the spectrum of the second ICA-separated signal (Figure 7d), the BPFO frequency component is enhanced, while the BPFI amplitude remains nearly unchanged. This indicates that the two characteristic frequencies associated with the inner and outer race faults have been effectively separated.

The source signal corresponding to the spectrum in Figure 7b can thus be identified with the inner race fault of the right bearing, whereas the signal in Figure 7d corresponds to the outer race fault of the left bearing. The indicator values for both separated source signals were increased and clearly decoupled compared to those obtained for the original signals.

Summarizing after ICA decomposition, two distinct source signals are obtained: one dominated by BPFI (inner race fault) and the other by BPFO (outer race fault). This clearly indicates that ICA is capable of separating overlapping fault signatures—a task where traditional envelope analysis or FFT-based methods typically fail.

### 5.4. Distribution Analysis of Fault Indicators

To obtain the distributions of the fault identification indicator values, the full vibration signal database (training dataset) was used. For each signal, indicator values were computed in the same manner as in the examples described above.

**Figure 8:**
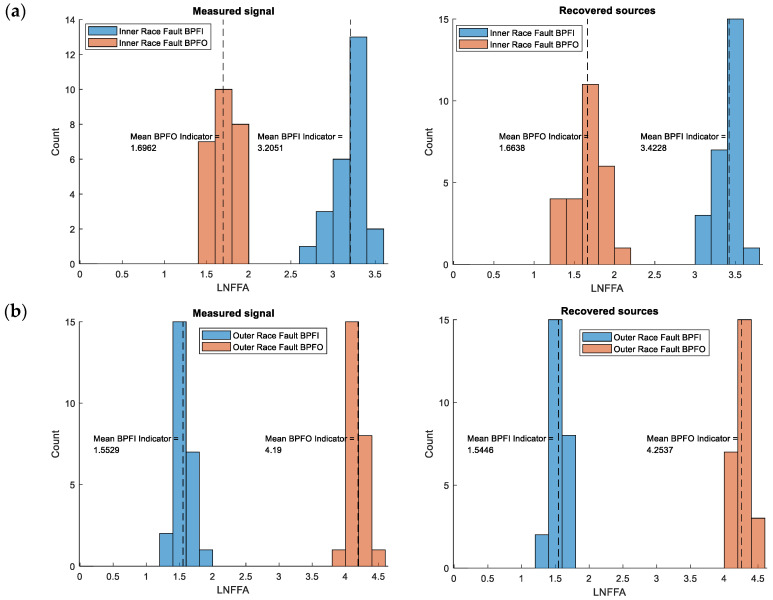
Comparison of LNFFA indicator distributions for single-fault conditions: (**a**) inner race fault of the left bearing, (**b**) outer race fault of the right bearing (measured signals—left histograms; ICA-separated signals—right histograms).

**Figure 9:**
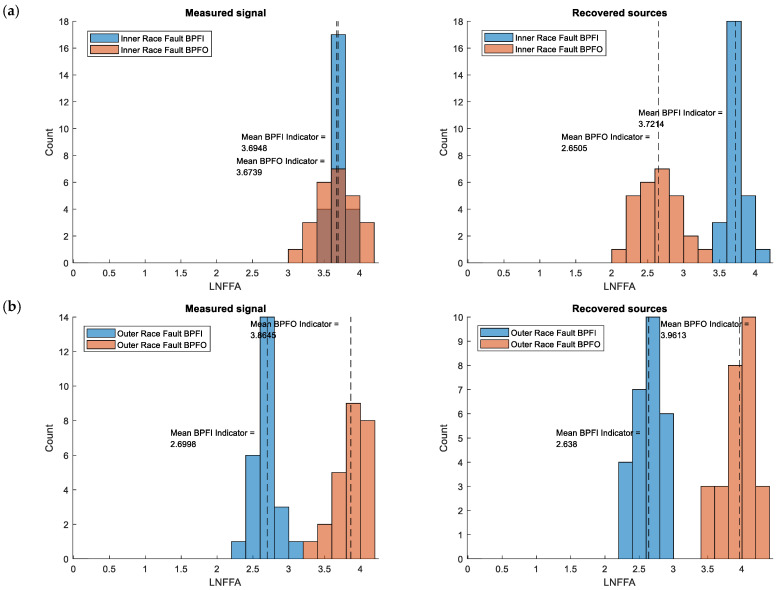
Comparison of LNFFA indicator distributions for simultaneous fault conditions: (**a**) inner race fault, (**b**) outer race fault.

The histograms in Figure 8 and Figure 9 show the distributions of the identification indicators corresponding to inner and outer race faults for all three fault conditions. For the single-fault cases and ICA-separated signals (Figure 8), there is a visible enhancement of the indicator corresponding to the specific fault and a greater separation between the indicator distributions. The indicator distribution associated with the fault shifts toward higher values, while the other indicator shifts toward lower values.

This effect becomes even more pronounced in the case of simultaneous faults. The distributions of the BPFI indicator for the original signals (Figure 9) overlap, whereas for the ICA-separated signals they are clearly separated. A similar, though less distinct, trend is observed for the BPFO indicator.

The vertical lines in the figures indicate the mean indicator values. As seen, the differences between mean values are greater for the ICA-separated signals than for the original ones.

The enhancement of the fault indicator corresponding to a given defect increases the probability of correct fault detection. Moreover, the separation of characteristic fault frequencies is advantageous when using fault-oriented indicators or composite indicators, such as the LRFA defined in Section 3. In such cases, the LRFA indicator would fail for original signals under compound fault conditions, as its value would correspond to normal operation (no fault). However, when applied to the ICA-separated signals, the same indicator correctly identifies both faults. Both of these effects—enhancement and separation of fault indicators—are therefore beneficial from the perspective of fault identifiability in vibration-based diagnostics.

Summarizing the results for ICA-separated signals, the histograms reveal increased separation between the distributions of BPFI and BPFO indicators. The mean indicator values (marked by vertical lines) are more distinct, confirming that ICA reduces the overlap between healthy and faulty conditions. In particular, under compound fault conditions, the ICA-processed signals exhibit a clear bimodal separation corresponding to the two fault types, whereas the original signals show a single overlapping distribution.

This statistical enhancement suggests that the ICA preprocessing stage significantly improves the sensitivity and specificity of fault detection methods based on characteristic frequency indicators, such as the LRFA and LNFFA, introduced earlier.

### 5.5. Comparative Analysis and Discussion

Table 1 summarizes the comparative results of the LNFFA indicator calculated for both the original vibration signals (after standard envelope analysis) and the ICA-separated source signals. The data encompass four experimental conditions: single inner race fault, single outer race fault, and two compound fault cases.

The comparison clearly demonstrates that applying ICA prior to envelope analysis leads to a significant enhancement in fault feature isolation** and improved diagnostic sensitivity. Consider the following, for example:

- In the single inner race fault scenario, the mean BPFI indicator increased from 3.21 to 3.42 after ICA, while the irrelevant BPFO component decreased from 1.70 to 1.66.
- In the single outer race fault scenario, the BPFO indicator rose from 4.19 to 4.25, while the unrelated BPFI component remained almost unchanged (1.55 to 1.54).
- For compound fault conditions, ICA effectively decoupled overlapping fault signatures:
- In the inner-dominant compound case, the difference between BPFI and BPFO indicators increased from Δ = −0.04 (before ICA) to Δ = +1.07 (after ICA), indicating a clear separation between fault-related spectral features.
- Similarly, in the outer-dominant compound case, BPFO increased from 3.86 to3.96, while BPFI remained stable (2.69 to 2.64), confirming selective enhancement of the relevant defect frequency.

Additionally, the standard deviations of the indicators decreased across all test cases (e.g., from 0.17 to 0.14 for BPFI in the inner race fault), which demonstrates that the ICA preprocessing yields more stable and repeatable diagnostic results.

#### 5.5.1. Comparison with Traditional FFT-Based Diagnostics

While traditional FFT analysis is effective in identifying steady-state harmonic content, it lacks the ability to distinguish overlapping fault frequencies, particularly under compound fault conditions. In our experiment, the envelope spectra of the raw signals exhibited simultaneous presence of BPFI and BPFO components, leading to ambiguity in fault identification. ICA resolves this limitation by performing blind source separation, thus revealing independent fault-related components without requiring prior knowledge of system dynamics or sensor placement.

#### 5.5.2. Relevance to Machine Learning and AI-Based Monitoring Systems

Recent studies have integrated deep learning models—such as CNNs and LSTMs—for automated bearing fault classification. However, such models rely heavily on large labeled datasets and extensive feature engineering. In contrast, the ICA method provides a physics-driven preprocessing framework that can improve the signal-to-noise ratio (SNR) and enhance feature separability prior to feeding data into machine learning pipelines. ICA can therefore be viewed as a hybrid bridge between model-driven signal analysis and data-driven AI systems.

#### 5.5.3. Comparative Diagnostic Value

The results indicate that ICA contributes complementary value to existing diagnostic frameworks:

- It enhances the effectiveness of traditional envelope analysis by isolating hidden fault components.
- It can serve as a feature extraction front-end for AI models, reducing the dependency on large training datasets.
- It preserves interpretability by maintaining the physical meaning of extracted components, unlike purely data-driven methods.

## 6. Conclusions

This manuscript presents the application of the ICA method to identify faults in rolling element bearings.

In the experimental study section, the results of comparing the distributions of fault identification indicators for simulated bearing defects were presented—both for the original measured vibration signals and for those obtained after ICA-based separation.

The comparative analysis confirms that integrating ICA into vibration-based diagnostics offers quantitative and qualitative improvements over conventional approaches. It not only amplifies the diagnostic indicators of bearing faults but also enables reliable discrimination under compound defect conditions, where traditional FFT and envelope analysis fail. Moreover, ICA can be seamlessly combined with machine learning pipelines to create hybrid intelligent monitoring systems that are both interpretable and data-efficient. Due to its high computational efficiency and numerical stability, the method has strong potential for implementation in real-time condition monitoring systems and industrial Internet of Things (IIoT) applications. Future research will focus on extending the methodology to a broader range of bearing faults, including ball and cage defects, and evaluating its performance under varying load and speed conditions. Moreover, integrating the ICA framework with adaptive filtering or machine learning classifiers will be explored to further improve robustness and automatic fault recognition in real-world environments.

## Figures and Tables

**Table 1:** Summary of comparative advantages.

Method	Strengths	Limitations	Relative Advantage of ICA
FFT/PSD Analysis	Clear visualization of harmonics	Ineffective for nonstationary or compound faults	ICA separates overlapping spectral features
Envelope Analysis	Sensitive to local impacts	Susceptible to masking and cross-modulation	ICA enhances envelope modulation contrast
ML/DL-based Systems	High automation, adaptive learning	Require large datasets, poor interpretability	ICA improves SNR and feature clarity for training
**ICA and Envelope Analysis (Proposed)**	Source separation, improved indicator sensitivity, model-independent	Computationally moderate, assumes linear mixtures	Provides hybrid diagnostic enhancement with classical and AI methods

## Data Availability

The data presented in this study are available on request from the corresponding author.

## Abbreviations

The following abbreviations are used in this manuscript:

REBs	Rolling Element Bearings
BSS	Blind Signal Separation
ICA	Independent Component Analysis
BPFI	Ball Pass Frequency on Inner race
BPFO	Ball Pass Frequency on Outer race
FTF	Fundamental Train Frequency (cage speed)
BSF	Ball Spin Frequency
LRFA	Log Ratio of Fault Amplitude
NAFF	Normalized Amplitude of Fault Frequency
LNFFA	Locally Normalized Fault Frequency Amplitude
SIM	Standard Identification Methods
LSTM	Long Short-Term Memory
